# Elephant Presence Shapes Butterfly Communities in an East African Coastal Forest

**DOI:** 10.1002/ece3.74532

**Published:** 2026-11-04

**Authors:** Jan Christian Habel, Thomas Schmitt, Maria M. Fungomeli, Mike Teucher, Jonas Eberle, Maximilian Hanusch, Werner Ulrich

**Affiliations:** ^1^ Evolutionary Zoology, Department of Environment and Biodiversity University of Salzburg Salzburg Austria; ^2^ Senckenberg German Entomological Institute Müncheberg Germany; ^3^ Entomology and Biogeography, Institute of Biochemistry and Biology, Faculty of Science University of Potsdam Potsdam Germany; ^4^ Coastal Forests Conservation Unit, Centre for Biodiversity National Museums of Kenya Nairobi Kenya; ^5^ Department of Geoecology, Institute of Geosciences and Geography Martin Luther University Halle‐Wittenberg Halle (Saale) Germany; ^6^ Department of Landscape Ecology and Vegetation Science, Institute of Landscape and Plant Ecology University of Hohenheim Stuttgart Germany; ^7^ Department of Ecology and Biogeography Nicolaus Copernicus University Toruń Poland

**Keywords:** butterfly diversity, East African coastal forest, habitat disturbance, habitat parameters, megaherbivores, primary forest, secondary forest, traits

## Abstract

Habitat disturbances shape the diversity and composition of plant and animal species. It is important to distinguish between natural disturbances caused by megaherbivores and anthropogenic disturbances caused by human activity, as different disturbance regimes differently affect biodiversity. In this study, we analyse butterfly diversity and community structure in three forest areas with different habitat disturbances in the Arabuko Sokoke dry coastal forest of south‐eastern Kenya. We assessed butterfly diversity with bait‐traps in primary forest with and without elephants, and in secondary forest characterised by previous human use and where elephants are currently present. In total, we activated 24 traps, eight traps per forest type. We found differences in the butterfly diversity and community structure depending on forest type and disturbance regime. The rather dense primary forest harboured a considerably higher proportion of dark‐coloured forest butterfly species than the secondary forest, particularly in areas with elephant disturbance. The secondary forest has higher butterfly species richness than the primary forest due to the occurrence of savannah species. Correspondence analyses clearly separated the three forest types with respect to community composition. Species occurring also in anthropogenic habitats were significantly more abundant in the secondary forest than in the two primary forest types. Our findings underline that forest type and disturbance regime shape butterfly diversity and community structure. Our study underlines that the effects of natural disturbances and human‐induced disturbances on butterfly diversity differ fundamentally. Natural disturbances such as by elephant activities seem to promote typical forest butterflies.

## Graphical abstract

Correspondence analysis of sites (a) and species (b) separated the three forest types (24 traps: yellow dots: sec + e, red: prim‐e, blue: prim + e) and the butterfly families (30 species).

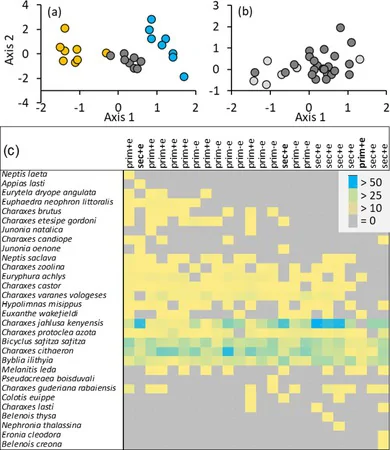

## 1 Introduction

Tropical forest ecosystems harbour a large proportion of the global biodiversity (Mittermeier et al. 2011). Deforestation and forest degradation negatively impact these forest ecosystems and their habitat quality, with negative effects on their plant‐ and animal species diversity (Newmark and McNeally 2018; Teucher et al. 2020). In East African forests, human disturbance includes selective logging, large‐scale clearing, as well as the expansion of cities (Aleman et al. 2017; Fungomeli et al. 2025). As a result, most of the remaining forests in East Africa are now in very poor condition or are secondary forests (Barlow, Gardner, et al. 2007).

Apart from habitat disturbances made by human beings, there are also natural agents that contribute to fundamental disruption and restructuring of forests. Mega‐herbivores such as elephants are known to act as ecosystem engineers shaping ecosystem structures and thus respective plant and animal species composition (Jones et al. 1994; Fritz 2017; Viljur et al. 2022). Such ecosystem engineers trigger natural vegetation succession and subsequently biodiversity sensu lato (Jones et al. 1997; Turner et al. 1997; Barlow, Gardner, et al. 2007; Castillo‐Campos et al. 2008; Espinosa‐Palomeque et al. 2020). In general, human disturbances, such as selective felling or clear‐cutting of a forest followed by regrowth, usually resulting in a decrease of habitat heterogeneity; In contrast, natural disturbances are generally more random and promote habitat heterogeneity and subsequently support biodiversity (Viljur et al. 2022).

Studies on the effects of habitat disturbance on biodiversity showed varying results (Barlow, Gardner, et al. 2007; Castillo‐Campos et al. 2008; Espinosa‐Palomeque et al. 2020; Turner et al. 1997). Habitat disturbances may negatively affect specific species and ecosystems (Jachmann and Bell 1984; Maicher et al. 2020) but may also increase habitat heterogeneity and subsequently promote the overall species diversity (Maicher et al. 2020; Samways and Kreuzinger 2001; Samways and Grant 2008). For example, stable primary forests provide complex filter regimes permeable only for species with a specific set of traits. Disturbances might cause declines in species richness but might also perforate these filters allowing some species to enter, which lack these forest‐specific traits (Durães et al. 2013; Barrett et al. 2023). Therefore, the study of altered filter regimes after disturbance should ideally be accompanied by the comparison of species trait expression. Studies on butterflies showed that the dispersal of typical forest butterfly species is rather restricted compared with those for open savannah species (Waltert et al. 2011). Butterfly wing coloration is often darker in species preferably found in forest ecosystems compared with butterfly species mainly occurring in open savannahs (Xing et al. 2016).

In this study, we test for effects of natural and human‐made forest disturbances on species diversity and community structure of butterflies in the Arabuko Sokoke coastal forest in southern Kenya. This forest represents one of the largest remnants of the East African dry coastal forest and has a long history of the use of forest resources by humans (Muriithi and Kenyon 2002). Thus, different forest types such as primary and secondary forest exist there. In parallel, in a fraction of the Arabuko Sokoke coastal forest there is a viable population of elephants, whose population size is constantly growing (Banks et al. 2010) and which are strongly shaping the vegetation structure (Habel et al. 2017). These elephants are kept out of part of the primary forest by an electric fence. This creates a unique exclosure situation and forms the prerequisite for the following forest situations: (1) Secondary forest with elephants, (2) Primary forest with elephants, (3) Primary forest without elephants.

We analysed butterfly (Papilionoidea) species richness and species community structure in these three forest types. Most butterfly species need specific habitat structures and rely on specific larval food plants for successful development (Boggs et al. 2003). As butterflies are highly sensitive to changes in the environment, they are often used as bioindicators (Brown and Freitas 2006). We expect that differences in habitat structures and plant composition due to past and current disturbances shape butterfly abundance, diversity and community composition (e.g., Moranz et al. 2012). Butterfly observations were conducted with bait traps. For each of the butterfly species caught with a trap, we compiled data on species‐specific ecological traits, such as habitat preferences and specialisation, morphology, and the distribution ranges of these species. With this information, we here investigate potential correlations between butterfly ecology and their reaction to habitat disturbance. Based on the data and results obtained, we ask the following research questions:

1. Does butterfly diversity and species composition differ depending on forest use and disturbance regimes?
2. Does the response of species to forest management and disturbance regimes depend on their ecological and behavioural performance?
3. What conclusions can be drawn from our findings regarding the protection of tropical forests subject to different types of disturbance?

## 2 Material and Methods

### 2.1 Study Area

The Arabuko Sokoke forest is a 416 km^2^ remnant of the East African dry coastal forest (Arabuko‐Sokoke Forest Management Team [ASFMT] 2002) (see Figure 1). The heterogeneous geology of coastal Kenya created the prerequisites for the development of various forest types, which today form a mosaic of different forest ecosystems (Muriithi and Kenyon 2002). In this study we focus on a single forest type, the Mixed forest, which grows on Pleistocene sands of aeolian origin (Karanja 1982). Mixed forest is dominated by the tree species *Afzelia quanzensis* Welw., *Hymenaea verrucosa* Gaertn., *Combretum schumannii* Engl., *Manilkara sansibarensis* (Engl.) Dubard, and *Encephalartos hildebrandtii* A. Braun & CD Bouché (Robertson and Luke 1993; Fungomeli et al. 2020). The Arabuko‐Sokoke forest has a very long history of land‐use (mining of siliceous sands, selective logging, removal of dead wood and charcoal production; Habel et al. 2017). A section of the forest (Mida area) was deforested during the 1920s during settlement. With the forest being placed under protection in the late 1970s (Mutangah and Mwaura 1992; Robertson and Luke 1993; Githitho 2021), the forest regenerated on these areas (at least since the early 1980s, the official end of commercial logging) (Fanshawe 1995).

**FIGURE 1:**
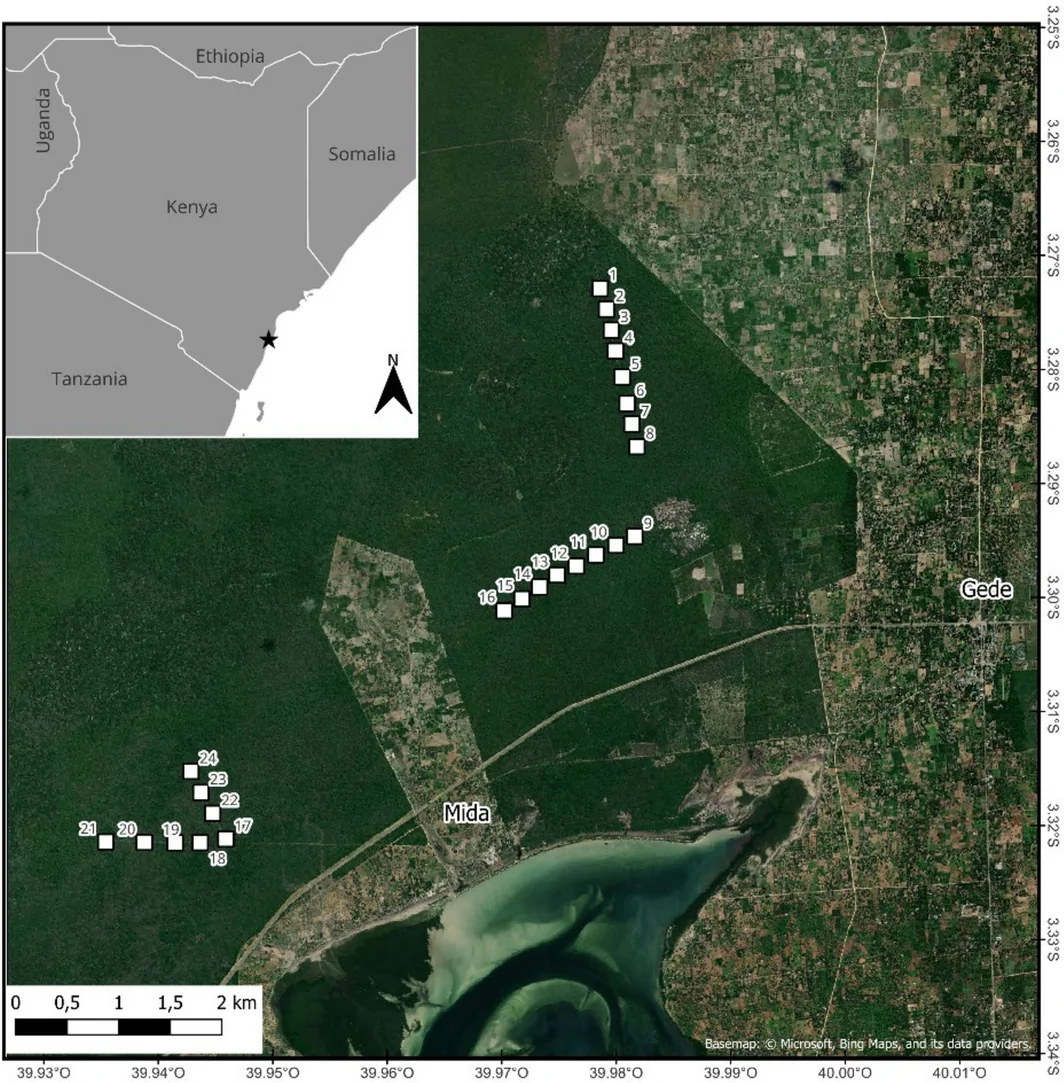
Study area in Kenya (star in small inlet map) and the locations of the 24 bait traps (white squares in large map) in the three different forest types, primary forest with elephants (traps 1–8), primary forest without elephants (traps 9–16) and secondary forest with elephants (traps 17–24). Numbers coincide with the numbering used throughout this article.

In addition to these human‐caused disturbances in the forest (such as in Mida area), there also exist disturbances caused by megaherbivores, for example, the constantly growing elephant population. These elephants strongly shape the vegetation structure and thus the composition of plant (and animal) species (Banks et al. 2010). These disturbances caused by elephants are randomly distributed throughout the forest ecosystem, thereby creating small‐scale heterogeneity (although disturbances caused by elephants in Arabuko Sokoke are concentrated around water sources and thus occupy a special position; for this reason, however, they were deliberately not included in the sampling here). The elephant population is excluded from parts of the forest (about 4% of the total area of Arabuko Sokoke forest) by an electric fence since about 20 years (Kinzer and Jackson 2009). Subsequently, different types of forest and disturbance regimes exist, which are derived from recent and historical activities: (1) Secondary forest after human disturbance and currently with elephants (sec + e); (2) Primary forest with elephants (prim + e) and (3) Primary forest without elephant and anthropogenic disturbances (prim‐e) (see Figure 1). We assume, that the diverging land‐use histories and differences in the current forest disturbance regimes shape forest structure, vegetation and animal communities and populations.

### 2.2 Recording of Butterflies

From 12 February 2025 to 20 February 2025 (9 days during the dry season) the butterfly fauna was recorded using bait traps (DC022 Pop‐up Butterfly bait trap, BugDorm). Bait traps are particularly suitable in tropical forest ecosystems to study butterflies, as many of them are attracted to rotten, that is, fermented fruits (Freitas et al. 2014). The bait consisted of overripe bananas, sugar, yeast, water and palm wine (for the exact recipe, see Habel et al. 2022). We ran eight traps in parallel in each of the three forest types (24 in total) and checked them daily between 9 AM and 5 PM in the same order. Thus, trap inspections were standardised to a fixed time each day. All traps were controlled after about 24 h (1 day and 1 night) and were activated eight times.

Traps were placed in typical forest surroundings. To guarantee spatial independence, distances between individual traps were always > 200 m. Previous studies conducted on identical taxa and in the region indicate dispersal range of about 10–120 m (see Habel et al. 2026). The traps were placed in mostly shaded but rather open areas at a height of 1.5 m (e.g., under a tree) to ensure that butterflies can easily access traps but also not suffer from heat and sun exposure. All selected trap locations were representative of the vegetation structure of mixed forest, to guarantee the comparability of the sampled butterfly diversities.

Most species could be examined and identified directly in the field. Photographs were taken of some species that are a little more difficult to identify, such as representatives of the family Pieridae and of the Nymphalid genus *Neptis*. Hereby we have taken photographs from the upper and underside of the wing of the respective individual and subsequently identified the species using appropriate literature (e.g., Larsen 1991). In this way, all the species caught in the traps could be identified without any difficulty. Among the species we recorded, there were no critical taxa that would, for example, have required a genital analysis for species identification (e.g., representatives of the family Lycaenidae). The complete list of species and number of individuals collected in the respective traps is given in Table S1. A selection of photographs of some species that we caught with our traps is compiled in Appendix S2.

### 2.3 Traits

Ecological traits, species distributions and morphology strongly determine species' responses to habitat changes (i.e., habitat disturbances) (Birkhofer et al. 2017). Thus, we compiled various traits for each butterfly species caught. Of central relevance is the evaluation of ecological performance. For this purpose, the degree of specialisation of the species during the larval stage was considered; we classified the species into monophagous (one plant species/genus), oligophagous (various genera within one family) and polyphagous (multiple families). This information is available for most butterfly species of Kenya and has been compiled from various literature sources, mainly from Larsen (1991). Disturbances in a forest can lead to fundamental vegetational changes across the different herbaceous layers, and thus specific groups of plants might profit or might be disadvantaged from various kinds of forest disturbances (Onaindia et al. 2004). Thus, we classified the larval diet into the following broad vegetation types: Mainly grasses and herbs, mixed and mainly bushes and trees. Information was compiled from Larsen (1991).

Furthermore, we considered the overall habitat preferences for each species, grouped into typical forest dwellers, species associated with woodlands and shrublands in savannahs, that is, more open and anthropogenic habitats (e.g., gardens, plantations), and ubiquitous species without clear preferences. Geographical distribution can also correlate with the degree of specialisation of the species in question, from local endemics to globally distributed species (Wardhaugh 2014). We have therefore grouped the species into the following distribution categories: Local (endemic to East African coastal forests), regional (restricted to eastern and southern Africa), continental (also found elsewhere in Africa or even beyond). Information on the larval food plant, habitat preference and distribution were compiled from Larsen (1991) and Habel et al. (2018).

Specific habitat resources, such as water, are of relevance for the occurrence of many butterfly species (Zhang et al. 2019). Thus, we classified all butterflies caught following the water index (arid, intermediate, not in arid habitats). Furthermore, we considered various key parameters on forest structures and habitat integrity that might be particularly relevant (especially for typical forest butterflies) (Attiwilli et al. 2022). For this purpose, we used the tree association index (does not need trees, needs trees, only in forests) and the savannah and hemeroby index (Hill et al. 2002); savannah association was classified into (1) not occurring in savannah, (2) mainly outside savannahs, (3) occurring in savannahs and forests and (4) mainly in savannahs. Hemeroby quantifies the anthropogenic influences on habitats (Jalas 1955; Hill et al. 2002). A low hemeroby score indicates a species' strong association with natural habitats, while a high score reflects a greater reliance on anthropogenic environments. Based on their hemeroby scores, species were classified into four categories: (1) occurring exclusively in natural habitats, (2) tolerating disturbed natural habitats, (3) inhabiting both natural and anthropogenic habitats and (4) being more prevalent in anthropogenic environments than in natural ones (Larsen 1991; Schmitt et al. 2020, 2021).

We also considered information about the morphology of the butterflies caught, as previous studies showed that habitat structure filters species according to their morphology (Xing et al. 2016). In doing so, we compiled information about wing span from Larsen (1991). And we also classified the butterflies according wing coloration, which was measured using the micaToolbox. We grouped the species into three classes: dark, medium, bright. All classifications for all species are given in Table S2.

### 2.4 Statistics

We used individual‐based rarefaction to compare species numbers among the three habitat types and estimated total richness using iChao1 (Hsieh et al. 2016). Differences in mean values and count data were assessed using *Z*‐tests with bootstrapped standard deviations (9999 samples) and using parametric contingency *χ* ^*2*^ tests. Evenness was assessed from the exponential Pielou index *J* = e*H*/*S* with *H* being the Shannon diversity and *S* the total number of observed species. Estimates of standard errors in iChao and *J* came from 9999 bootstrap samples. Abundances were estimated from the total number of records of each species.

Differences in species composition among the investigated sites were assessed using correspondence analysis (seriation) of the species 00D7 trap abundance matrix in R (v. 4.2.2; R Core Team 2024) using ade4 (v. 1.7‐23; Dray and Dufour 2007). A plot representing the relative abundance of species by sites was used to illustrate the organisation of butterfly communities. Therefore, sites and species were reordered along the plot axes according to the first correspondence axis.

*Charaxes* butterflies are highly mobile and can fly several km (Habel et al., unpublished data). Trap distances across the three forest types ranged between 1 km and > 10 km. Therefore, spatial autocorrelation among the three forest types might be an issue when comparing species richness patterns. We could not assess whether a species caught in a given forest also reproduces there or only came to feed. Therefore, our richness estimates and community comparisons naturally refer to residents and tourists, potentially including multiple counts of individuals.

## 3 Results

In total, we recorded 30 butterfly species, 23 species in the primary forest with elephants (prim + e), 19 species in the primary forest without elephants (prim‐e) and 25 species in the secondary forest with elephants (sec + e) (Table 1, Figure 2). iChao1 richness estimates indicated a total of 26 species in prim + e, 39 species in sec + e and 28 species in prim‐e (Figure 2a). Rarefaction curves of prim + e and sec + e intersected, in line with a fast accumulation of richness in prim + e and therefore a higher evenness compared to sec + e. These differences were mainly due to the six Pieridae species, five of which were caught exclusively in sec + e (Figure 2c). The Pielou evenness of sec + e was *J* = 0.21 ± 0.03, of prim + e it was *J* = 0.38 ± 0.03 and 0.34 ± 0.02 for prim‐e, making sec + e significantly less even (*p* (*t*) < 0.05) than both primary forests that did not significantly differ in richness and evenness at the 95% error level. Species overlap among the three study sites was 53.3% (Figure 2b). This percentage increased to 66.7% when excluding Pieridae that nearly exclusively occurred in sec + e (Figure 2d). Only the forest specialist *Pseudacraea boisduvali* (Nymphalidae) was trapped exclusively in prim‐e.

**TABLE 1:** Numbers of records and species of major species trait categories in three forest types (prim + e, prim‐e and sec + e). In bold are given contingency table significances among groups at *p* < 0.01. Mean wing lengths are given with standard errors.

Variable	Records			Species		
	prim + e	prim − e	sec + e	prim + e	prim − e	sec + e
Total numbers	782	964	1100	23	19	25
	*Family*					
Nymphalidae	781	964	1091	22	19	19
Pieridae	1	0	9	1	0	6
	*Phagy*					
Monophagous	3	1	4	1	1	2
Oligophagous	276	302	393	7	6	9
Polyphagous	503	661	703	16	13	16
	*Colour class*					
Dark	**566**	**567**	**326**	10	8	8
Medium	**211**	**382**	**763**	12	10	11
Light	**5**	**15**	**11**	1	1	6
	*Habitat*					
Forest	**365**	**384**	**157**	9	8	8
Ubiquitous	**338**	**353**	**419**	8	8	10
Woodland	**79**	**227**	**524**	6	3	7
	*Distribution*					
Local	**406**	**380**	**445**	13	12	14
Regional	**3**	**0**	**2**	1	0	1
Continental	**373**	**584**	**653**	9	7	10
	*Wing length*					
Mean wing length	37.4 ± 2.7	39.7 ± 2.2	35.1 ± 2.5	—	—	—

**FIGURE 2:**
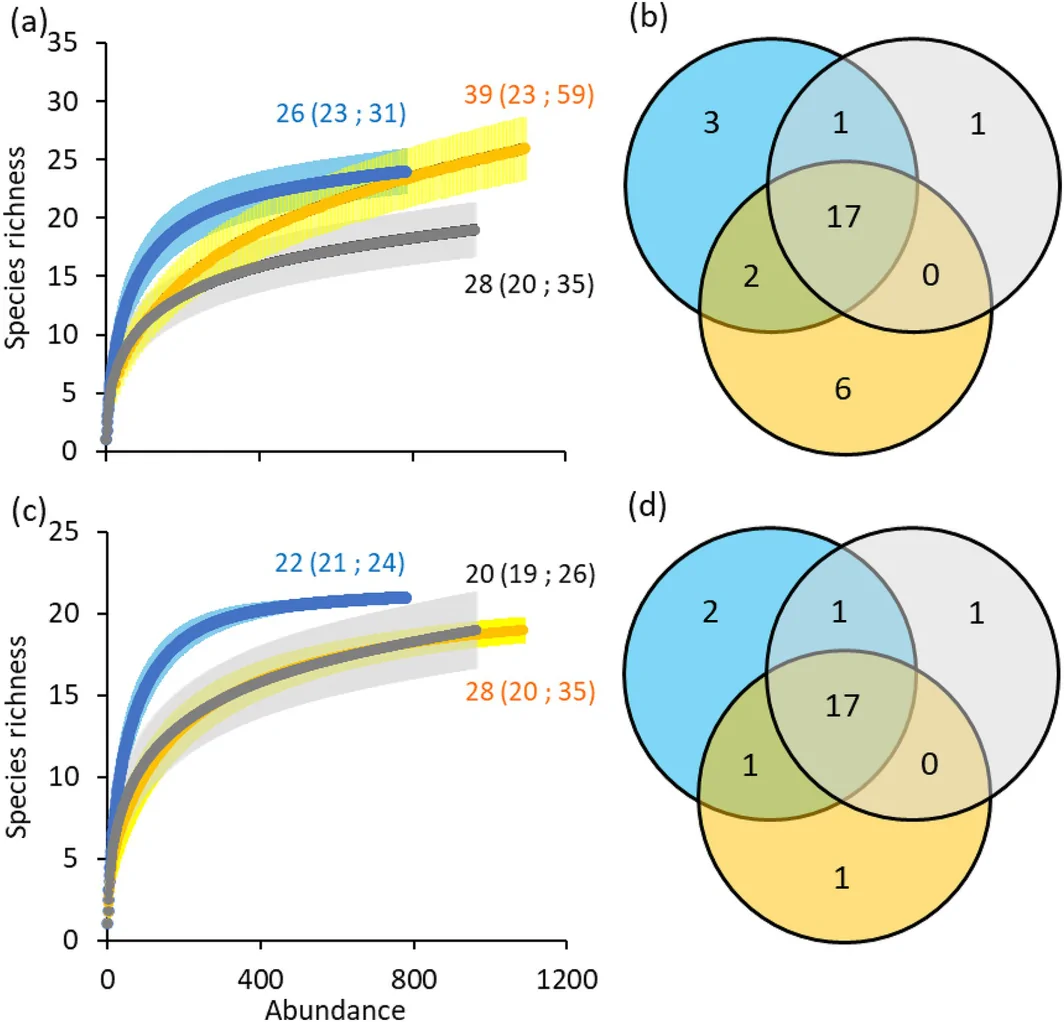
(a) Rarefaction curves (with 95% confidence limits) for butterflies from the primary forest with elephants (blue), the primary forest without elephants (grey) and the secondary forest with elephants (yellow). (a, b) All species, (c, d) Nymphalidae only. The numbers indicate the iChao1 richness estimates with bootstrapped lower and upper 95% confidence limits. (b) Venn diagram (colours as in a) showing the total numbers of species and the species overlap between the three habitat types.

Sample sizes were highest in sec + e (1,100 records), intermediate in prim‐e (964) and lowest in prim + e (782) (Table 1). The following species reached high abundances in all forest types and in total accounted in each of the three types for more than 50% of all records: four Charaxinae species *Charaxes protoclea* (Feisthamel, 1850), *C. varanes* (Cramer, 1764), *C. cithaeron* (C. & R. Felder, 1859) and *C. jahlusa* (Trimen, 1862), one Satyrinae, *Bicyclus safitza* (Westwood 1850) and one Biblidinae, *Byblia ilithyia* (Drury 1773) (Table S1). Of these, *C. cithaeron* was the only species more frequent in *prim‐e* than in the two other forest types; *C. varanes* was most common in prim + e; *B. ilithyia* and *C. jahlusa* were the dominant species in sec + e, while *C. protoclea* and *B. safitza* were more common in the two primary forest types than in the secondary forest (Table S1). Overlap (Figure 2b,d) and correspondence analyses (Figure 3) clearly separated the three forest types with respect to butterfly community composition.

**FIGURE 3:**
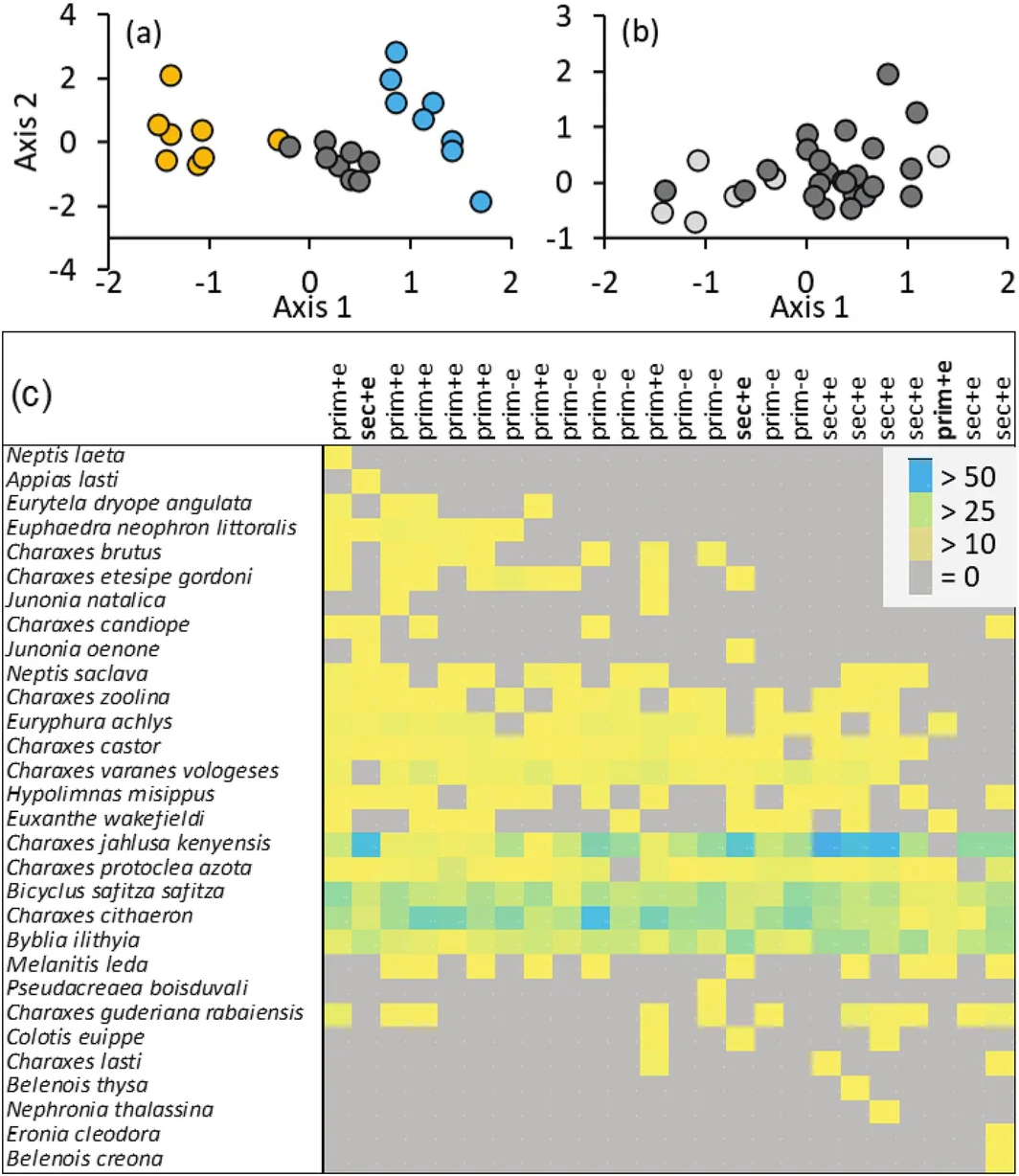
Correspondence analysis of sites (a) and species (b) separated the three forest types (24 traps: Yellow dots: Sec + e, grey: Prim‐e, blue: Prim + e) and the butterfly families (30 species: Light grey dots: six6 Pieridae species, dark grey: 24 Nymphalidae species) according to the first CA axis (44.0% variance explanation, the second axis explained 10.1% of variance). (c) Table plot of species communities and abundance classes (numbers of records) at the investigated sites in the three forest types. In (a) and (b) species and traps were arranged according to the first and second axes, in (c) according to the first axis of a correspondence analysis (seriation) of the community data. In (c) three outlying plots are marked in bold type.

These differences are reflected in the distribution of species traits, and functional trait composition significantly (at the 1% error level of contingency probabilities) differed among the three habitat types (Tables 1 and 2). More in detail, monophagous species were largely absent from the three habitat types, while polyphagous were the dominant group, but no significant difference was obtained among the habitat types (Table 1). Both primary forest types were dominated by darker colour types, while lighter coloured individuals were most common in sec + e. Species associated more with open landscapes (i.e., the categories wood‐ & shrubland, ubiquitous) were most common in (Table 1). In the latter, species of continental or even wider distribution dominated, while species restricted to eastern and southern Africa were more common in prim + e (Table 1). We did not find significant differences in average wing length among the forest types (*p*(*t*) > 0.1), but the largest average wing size was obtained in prim‐e (Table 1).

**TABLE 2:** Numbers of records and species of the hemeroby, tree, savannah, water and larval diet type index categories in three forest types (prim + e, prim‐e and sec + e). In bold are given contingency table significances among groups at *p* (*χ* ^2^) < 0.01.

Variable	Records			Species		
	prim + e	prim − e	sec + e	prim + e	prim − e	sec + e
	*Hemeroby*					
Natural habitats	**74**	**59**	**33**	4	4	4
Disturbed natural habitats	**406**	**602**	**663**	12	8	11
Natural and anthropogenic habitats	**293**	**298**	**393**	6	6	8
Common in anthropogenic habitats	**9**	**5**	**11**	1	1	2
	*Tree index*					
Does not need trees	**89**	**129**	**249**	6	5	8
Needs trees	**295**	**446**	**674**	5	4	6
Only in forests	**398**	**389**	**177**	12	10	11
	*Savannah index*					
No savannah	**50**	**31**	**9**	3	4	4
Mainly outside savannahs	**315**	**329**	**144**	8	5	7
Savannahs and forests	**114**	**84**	**68**	7	6	7
Mainly savannahs	**303**	**520**	**879**	5	4	7
	*Water index*					
Arid habitats	**90**	**134**	**238**	4	4	5
Intermediate	**586**	**765**	**822**	11	9	13
Not in arid habitats	**106**	**65**	**40**	8	6	7
	*Larval diet type index*					
Grasses and herbs	**271**	**289**	**385**	5	4	5
Trees and Shrubs	**39**	**13**	**14**	4	2	2
Both	**472**	**662**	**701**	14	13	18

Tree and savannah indices revealed contrary patterns, with savannah associated species being most common in sec + e (Table 2). Species of intermediate water index occurred similarly in all three forest types. Species associated with forests (even disturbed ones) were more common in prim + e and prim‐e than in sec + e (Table 2). Larval diets differed among the forest types, although this was only due to some species strictly associated with trees and shrubs (Table 2). Species occurring also in anthropogenic habitats were significantly more abundant in the sec + e forest than in both other forest types (Table 2).

## 4 Discussion

### 4.1 Butterfly Species Richness

The richness estimates obtained did not exceed 40 butterfly species, and rarefaction analyses revealed a high degree of saturation of species numbers after just a few days of data collection for the three forest types. Our study design thus was able to estimate the number of butterfly species with sufficient precision. The total number of reported species is similar to prior studies using the same technique conducted in other parts of East Africa (Habel et al. 2022) as well as from West Africa (Fermon et al. 2000, 2003, 2005) and from other parts of the world such as the Neotropics (DeVries et al. 2012; Freitas et al. 2014; Checa et al. 2019; Álvarez et al. 2021), South‐East Asia (Sari et al. 2025) and Europe (Jakubikova and Kadlec 2015). The comparatively low number of species assessed is due to the sampling method used here. Bait traps mainly catch butterflies that are attracted by rotten fruits (Freitas et al. 2014) and most of the butterfly diversity feeding on nectar sources are not attracted by the bait and thus not represented by this method. Thus, a large proportion of the individuals caught with our traps represent only a taxonomic fraction of the total butterfly diversity, most of them belonging to the family Nymphalidae (particularly the genus *Charaxes*).

Comparing the three different forest areas, butterfly diversity was highest in the secondary forest with elephant disturbance and lowest in the primary forest without elephants. The latter forest type contains herb, shrub and tree layer and the plant species composition significantly differ from the other forest areas (see also Robertson and Luke 1993; Abdallah 2017). Our results on the species richness in the different forest types are largely consistent with findings from other studies. For example, butterfly species richness was highest in disturbed forests, but abundance and diversity of endemic (i.e., forest specialist) butterflies were significantly higher in undisturbed forests as studied in Sulawesi (Indonesia) (Fermon et al. 2000, 2003, 2005; Veddeler et al. 2005; Koh 2007). A shadier forest climax stage is mostly characterised by a rather homogeneous vegetation and comparatively low plant species diversity (Viljur et al. 2022). In contrast, natural forest disturbance occurs randomly and enhances plant species richness (Rosenfield et al. 2023). This increase in plant diversity positively affects insect diversity (Basset et al. 1998; Wood and Gillman 1998). Disturbances in forest ecosystems may also change microclimatic conditions and promotes habitat heterogeneity as well as light exposure which are key factors for the presence of many insects (Wood and Gillman 1998; Viljur et al. 2022). Numerous insects such as butterflies preferably spend time at sunny spots during the adult stage and successful larval development often rely on specific microclimatic conditions (Hinneberg et al. 2025).

Anthropogenic disturbances and natural disturbances result in very different habitat structures. Human‐induced disturbances, for example, often create very similar habitat conditions, as selective logging and large‐scale disturbances (such as the clearing of large areas of forest) usually result in a homogeneous situation (Viljur et al. 2022). In contrast, natural disturbances are usually random and occur on a small scale; as such, they promote diversity in niche availability and thus provide habitats for a great many organisms (Viljur et al. 2022).

### 4.2 Community Structure Among Forest Types

Despite differences in vegetation, the butterfly communities overlapped considerably in their composition among the three forest types. Even in the secondary forest, only 6 out of 25 species were not found in the other forest types, that is, five of the six Pieridae species as well as the Nymphalid *Junonia oenone* (Linnaeus 1758). Correspondence analysis clearly separated the forest types according to family membership. Most of the Pierids are generalists and predominantly found in more open ecosystems beyond closed forests; *J. oenone* even is a typical savannah species (Larsen 1991). Vice versa, we recorded typical forest species such as *Charaxes brutus* (Cramer 1779), *Charaxes etesipe* (Godart 1824) and *P. boisduvali* (Doubleday 1845) only or predominantly in primary forest.

Differences in community structure were even more obvious when including species abundances with prim + e and sec + e being the most distinct. For instance, the savannah species *B. ilithyia* and the wood‐ and bushland species *C. jahlusa* (Larsen 1991) were most dominant in the secondary forest, while the forest specialists *Euxanthe wakefieldi* (Ward 1873), *E. achlys* (Hopffer 1855), and *C. cithaeron* (Larsen 1991) occurred in much higher abundances in the primary forest (with and without elephants). Such differences in the butterfly community structure can also be influenced by potential stratification of species communities (see Fermon et al. 2003, 2005). Although the traps were set at the same height (close to the ground) in all three forest sites, the habitat structure could affect the stratification of species depending on the degree of forest disturbance. For example, typical canopy dwellers such as *E. wakefieldi* and *C. etesipe* might be more likely to be attracted to a bait trap located on the ground in a disturbed forest than in an intact forest.

Our results underline the long‐lasting effects of human disturbance, well reflected in the higher proportion of disturbance indicators in the secondary forest compared with the two types of primary forest. Although the secondary forest is regaining many of the typical forest butterfly species from the adjoining primary forests, some of the forest specialist butterflies were not observed there or at least at considerably lower densities, hence supporting the non‐substitutable value of intact primary forests. Previous studies in the Arabuko Sokoke forest based on line transects showed similar trends, with typical forest butterflies mainly inside intact forests (Habel et al. 2018). Similar findings were also made in the cloud forests of the Taita Hills (Kenya) (Schmitt et al. 2020), and in the Amazon rainforest (Barlow, Overal, et al. 2007). These studies and our own data underline the high conservation value of primary forests for many specialist species.

### 4.3 Functional Traits and Forest Habitat Structures

The higher species richness in disturbed tropical forests mostly results from the strong increase of taxa typical for more open landscapes, which overcompensates for the loss of typical forest species. This finding is in line with other studies on butterflies conducted in forest ecosystems (Basset et al. 1998). This trend is also reflected in the habitat selection and attachment to trees of the individual species. 32.9% of the species and 22.6% of the individuals in the secondary forest are not in need of trees; in the primary forest, it was 25.0% (species) and 12.5% (individuals). Hence, the typical forest species are more predominant in the primary forest, thereby underlining its considerably higher conservation value compared to the secondary forest. Furthermore, species with smaller distribution areas dominate in the primary forest. Typical forest species such as *C. brutus* , *C. etesipe* and *E. wakefieldi* were more common in the primary forest with elephant disturbance than in the primary forest without elephants. This is also well reflected in the percentage of individuals belonging to the truly specialised forest species, which was 14.3% in secondary forests, 38.8% in prim‐e and 46.7% in prim + e. This difference between the two primary forest types at first glance seems counterintuitive. However, elephants apparently have been a permanent and long‐lasting source of natural disturbance in the forests of Africa (Johnson et al. 2007), and most forest species are strongly adapted or even rely on these habitat disturbances.

Consequently, natural elephant disturbance and anthropogenic disturbance might have opposing influences on specialised forest butterfly species. The butterfly community found in the secondary forest was dominated by common generalist species found in open landscapes such as *Melanitis leda* (Linnaeus 1758), which might have been favoured by the higher availability of grasses, that is, its larval food plant (Larsen 1991), or even disturbance indicators like *Hypolimnas misippus* (Linnaeus 1764) (Larsen 1991). However, also some forest species apparently benefited from forest disturbance in general, thus the gliding species *Neptis saclava* (Boisduval 1833) which, due to its behaviour, prefers less dense forest structures, and *Charaxes guderiana* (Dewitz 1879), a species typical for the more open, hall‐like *Brachystegia* forests (Larsen 1991).

We found that comparatively more species with dark wings occurred in the denser forest, while those with lighter coloured wings were found more in the disturbed forests, with lightest colours observed in the secondary forest where tree cover was lowest. The influence of the environment on the dark coloration of organisms has been frequently studied and widely discussed. Studies show that the level of melanisation in individuals of the same species increases with altitude (Gloger's rule) (see Delhey 2019). The dependence of coloration on corresponding forest habitat structures has also been demonstrated. Studies on tropical butterflies have found positive correlations between forest density and wing darkness (Xing et al. 2016). This could be related to the butterflies' thermoregulation in habitats with varying temperatures and humidity (see Xing et al. 2016; Stelbrink et al. 2019). To unravel the ultimate causes behind these colour differences, further physiological and evolutionary studies are needed (Lopez et al. 2024).

## 5 Conclusions

Our results indicate that habitat disturbances caused by elephants and humans have different effects on butterfly species richness and assemblage composition. However, we note some limitations of our study design, particularly regarding landscape‐scale replication and temporal coverage. A combination of different observation methods with long‐term monitoring might return more quantitative and species‐specific answers about the differential influence of elephants and humans on the nectar feeding butterflies.

Despite these shortcomings, we can conclude that a primary forest promotes ecologically demanding forest species. While the effects of disturbances caused by elephants have already been extensively studied in savannah ecosystems, our study shows for the first time how megaherbivore activity impacts biodiversity in a forest ecosystem and that typical forest butterfly species still occur in forested areas shaped by significant natural disturbances (here by elephants).

## Author Contributions

**Jan Christian Habel:** conceptualization (equal), data curation (equal), investigation (equal), methodology (equal), project administration (equal), supervision (equal), validation (equal), writing – original draft (equal), writing – review and editing (equal). **Thomas Schmitt:** conceptualization (equal), methodology (equal), writing – original draft (equal), writing – review and editing (equal). **Maria M. Fungomeli:** data curation (equal), investigation (equal), writing – original draft (equal), writing – review and editing (equal). **Mike Teucher:** data curation (equal), investigation (equal), writing – original draft (equal), writing – review and editing (equal). **Jonas Eberle:** conceptualization (equal), data curation (equal), visualization (equal), writing – original draft (equal), writing – review and editing (equal). **Maximilian Hanusch:** investigation (equal), writing – review and editing (equal). **Werner Ulrich:** conceptualization (equal), data curation (equal), formal analysis (equal), software (equal), supervision (equal), validation (equal), visualization (equal), writing – original draft (equal), writing – review and editing (equal).

## Conflicts of Interest

The authors declare no conflicts of interest.

## Supporting information


**Table S1:** Records of butterfly species in three forest types of the Arabuko Sokoke coastal forest (prim + e: primary forest with elephants; prim − e: primary forest without elephants; sec + e: secondary forest with elephants).
**Table S2:** Ecological and morphological charcteristics of butterfly species in three forest types of the Arabuko Sokoke coastal forest.

**Appendix S2:** Elephant presence shapes butterfly communities in an East African coastal forest.

## Data Availability

All data will be freely online available as Supporting Information.
