# Supplementary material for: Elephant Presence Shapes Butterfly Communities in an East African Coastal Forest
Source: Ecol Evol. 2026 Nov 4;16(11):e74532. doi: 10.1002/ece3.74532 (PMC13733380; doi:10.1002/ece3.74532)
Supplement: Supplementary file 1 [file ECE3-16-e74532-s001.docx]

**Appendix 2:**

**Elephant presence shapes butterfly communities in an East African coastal forest**

**Pictures of butterflies captured in bait traps in the Arabuko Sokoke forest.**

*Bicyclus safitza* is a common Satyrinae found in almost all places with some shade.

*Byblia ilithyia* frequently visits bleeding trees. The species is widespread in a large variety of different habitats.

*Charaxes brutus* as all Charaxinae is strongly attracted by rotting fruits and therefore frequently encountered in bait traps.

*Charaxes candiope* is mostly a savannah species, but also occurs in forest habitats

*Charaxes varanes* is a generalist and widespread species often encountered in forest habitats.

*Charaxes zoolona* is the smallest member of this genus encountered in our traps.

*Euphaedra neophron* is often basking with open wings on sunny spots of forests, but also under small groups of trees.

*Euryphura achlys* is a typical forest species, mostly found flying close to the ground.

*Euxanthe wakefieldi* is a species mostly exclusively found in forests with low levels of human disturbance.

*Junonia natalica* is a widespread species in eastern and southern Africa found in forests and thickets of savannah habitats.

*Neptis saclava* is maybe the most common species of this genus in Africa. The butterflies are often gliding around trees.
